# *N*,*N′*,*N′′*-Tris(β-hydroxyethyl)hexahydro-1,3,5-triazine: MAK Value Documentation, addendum – Translation of the German version from 2023

**DOI:** 10.34865/mb471904kske10_3ad

**Published:** 2025-09-29

**Authors:** Andrea Hartwig, MAK Commission

**Affiliations:** 1 Institute of Applied Biosciences. Department of Food Chemistry and Toxicology. Karlsruhe Institute of Technology (KIT) Adenauerring 20a, Building 50.41 76131 Karlsruhe Germany; 2 Permanent Senate Commission for the Investigation of Health Hazards of Chemical Compounds in the Work Area. Deutsche Forschungsgemeinschaft, Kennedyallee 40, 53175 Bonn, Germany. Further information: Permanent Senate Commission for the Investigation of Health Hazards of Chemical Compounds in the Work Area | DFG

**Keywords:** N,N′,N′′-tris(β-hydroxyethyl)hexahydro-1,3,5-triazine, nose, larynx, trachea, irritation, formaldehyde releaser, carcinogenicity, germ cell mutagenicity, sensitization, hydrolysis

## Abstract

The German Senate Commission for the Investigation of Health Hazards of Chemical Compounds in the Work Area summarized and re-evaluated the data for *N,N′,N′′*-tris(β-hydroxyethyl)hexahydro-1,3,5-triazine [4719-04-4] with regard to its carcinogenicity and germ cell mutagenicity classifications, its ability to be absorbed through the skin and in order to derive an occupational exposure limit value (maximum concentration at the workplace, MAK value). Relevant studies were identified from a literature search and also unpublished study reports were used. *N,N′,N′′*-Tris(β-hydroxyethyl)hexahydro-1,3,5-triazine rapidly releases formaldehyde in dilute aqueous solution. The substance is highly irritating to corrosive to the skin and eyes of rabbits and to the upper respiratory tract, which is attributed to the formation of the irritating formaldehyde and 2-aminoethanol. There are no studies that investigated the carcinogenic effects of *N,N′,N′′*-tris(β-hydroxyethyl)hexahydro-1,3,5-triazine and its genotoxic potential in the upper respiratory tract or nose. Formaldehyde was classified in Carcinogen Category 4 because it causes tumours in nasal tissues at concentrations that exceed their detoxification capacity. As formaldehyde is released from *N,N′,N′′*-tris(β-hydroxyethyl)hexahydro-1,3,5-triazine, the substance could likewise be classified in Carcinogen Category 4. In a 28-day inhalation study in rats with an aerosol of *N,N′,N′′*-tris(β-hydroxyethyl)hexahydro-1,3,5-triazine, multifocal squamous metaplasia of the larynx and nose as well as degeneration of the bronchial epithelium were observed at the lowest concentration tested of 3 mg/m^3^ and above. Due to the severity of these effects, it is not possible to derive a NOAEC or a MAK value from this study. Moreover, a MAK value cannot be established in analogy to the MAK value set for gaseous formaldehyde because aerosol impaction aggravates the effects. The substance has thus been assigned to Carcinogen Category 2 with the footnote “Prerequisite for Category 4 in principle fulfilled, but insufficient data available for the establishment of a MAK or BAT value”. As there are no data on the systemic bioavailability of *N,N′,N′′*-tris(β-hydroxyethyl)hexahydro-1,3,5-triazine and formaldehyde released by hydrolysis in tissues, there is no experimental evidence that the formaldehyde reaches the germ cells. Therefore, *N,N′,N′′*-tris(β-hydroxyethyl)hexahydro-1,3,5-triazine has been classified in Category 3 B for germ cell mutagens. Skin contact is not expected to contribute significantly to systemic toxicity.

**Table:** 

**MAK value**	**–**
**Peak limitation**	**–**
	
**Absorption through the skin**	**–**
**Sensitization (2014)**	**Sh**
**Carcinogenicity (2022)**	**Category 2** ^a)^
**Prenatal toxicity**	**–**
**Germ cell mutagenicity (2022)**	**Category 3 B**
	
**BAT value**	**–**
	
CAS number	4719-04-4
Molar mass	219.3 g/mol
Vapour pressure	5 × 10^–8^ hPa at 25 °C (calculated) (Hartwig 2015, available in German only)

^a)^ prerequisite for Category 4 in principle fulfilled, but insufficient data available for the establishment of a MAK or BAT value

Note: releases formaldehyde

For *N*,*N′*,*N′′*-tris(β-hydroxyethyl)hexahydro-1,3,5-triazine there is documentation available from 1988 (Henschler 1991), an addendum on sensitization from 1995 (Greim 1998) and an addendum on all end points from 2015 (Hartwig 2015, available in German only). Previously, *N*,*N′*,*N′′*-tris(β-hydroxyethyl)hexahydro-1,3,5-triazine was assigned to Section II b of the List of MAK and BAT Values. Cited unpublished toxicological studies from companies have been made available to the Commission.

*N*,*N′*,*N′′*-Tris(β-hydroxyethyl)hexahydro-1,3,5-triazine releases formaldehyde in aqueous solution. This addendum has become necessary because a further hydrolysis study has become available. The data for the release of formaldehyde are re-evaluated here according to the current procedures of the Commission and the end point carcinogenicity is re-considered from this aspect.

## Toxicokinetics and Metabolism

### Hydrolysis

As is known from other structurally similar formaldehyde releasers, for example *N*,*N′*,*N′′-*tris(β-hydroxy**propyl**)hexahydro-1,3,5-triazine (Hartwig and MAK Commission 2025), the rate of hydrolysis of *N*,*N′*,*N′′-*tris(β-hydroxyethyl)hexahydro-1,3,5-triazine is dependent on the concentration of the solution, the pH and the temperature. The hydrolysis products are 2-aminoethanol (monoethanolamine) and formaldehyde.

The half-lives for the hydrolysis of *N*,*N′*,*N′′*-tris(β-hydroxyethyl)hexahydro-1,3,5-triazine at pH 7 and pH 9 are 50 and 302 days, respectively. At a roughly physiological pH of 7, the amount of formaldehyde released from *N*,*N′*,*N′′*-tris(β-hydroxyethyl)hexahydro-1,3,5-triazine (no other details) after one day is about 19% (Hartwig 2015).

In a hydrolysis study from 2010, *N*,*N′*,*N′′*-tris(β-hydroxyethyl)hexahydro-1,3,5-triazine decomposed immediately in a 0.1% aqueous solution at pH 4 and 37 °C to form 2-aminoethanol and formaldehyde (Hartwig 2015; RAC and SEAC 2020). Also at pH 7 and 37 °C, hydrolysis is extremely rapid (no other details; RAC and SEAC 2020).

In a test according to OECD Test Guideline 111 which has recently become available, a 79% *N*,*N′*,*N′′*-tris(β-hydroxyethyl)hexahydro-1,3,5-triazine solution was completely hydrolysed at 50 °C within 2 hours (BASF AG 2002).

*N*,*N′*,*N′′*-Tris(β-hydroxyethyl)hexahydro-1,3,5-triazine hydrolyses more rapidly at pH 8 than at pH 9.5 or 10.9. At 22 °C, the half-lives for these pH values were 32 seconds, 16 minutes and 3.4 hours, respectively (Hartwig 2015). It is likely that these data refer to a dilute solution that hydrolyses much more rapidly, as it is known from other similar formaldehyde releasers such as *N*,*N′*,*N′′*-tris(β-hydroxy**propyl**)hexahydro-1,3,5-triazine (Hartwig and MAK Commission 2025).

In metal-working fluid concentrate, incomplete hydrolysis of *N*,*N′*,*N′′*-tris(β-hydroxyethyl)hexahydro-1,3,5-triazine can be assumed. In water-mixed metal-working fluid, on the other hand, complete hydrolysis to formaldehyde and 2-aminoethanol is to be expected within a short time due to the strong dilution in aqueous solution (3% solutions). For this application, reference is therefore made to the MAK documentation or supplements on formaldehyde (Greim 2002; Hartwig 2014) and 2-aminoethanol (Greim 1999; Hartwig and MAK Commission 2018).

## Animal Experiments and in vitro Studies

### Subacute, subchronic and chronic toxicity

#### Inhalation

The documentation from 1988 describes a 5-week inhalation study in male and female Hartley guinea pigs exposed to aerosol mixtures of 0.15% or 1.5% *N*,*N′*,*N′′*-tris(β-hydroxyethyl)hexahydro-1,3,5-triazine and cutting oil in water (1:3 and 1:33, respectively). Swelling of the eyes and profuse lacrimation, mild pulmonary emphysema, occasional slight focal inflammation in the heart and kidneys, and the accumulation of eosinophilic cells in the spleen were observed (Henschler 1991).

In a 28-day inhalation study according to OECD Test Guideline 412, described in detail in the documentation from 2015, male and female Wistar rats were exposed nose-only for 6 hours a day, on 5 days per week to aerosols of *N*,*N′*,*N′′*-tris(β-hydroxyethyl)hexahydro-1,3,5-triazine at concentrations of 0, 3, 10, 30 or 100/50 mg/m^3^ (nominal concentrations: 6.1, 17.4, 50.6 or 172.4/86.2 mg/m^3^). At the lowest concentration of 3 mg/m^3^ and above, squamous metaplasia, erosion/ulcers, necrosis of U-shaped cartilage, hyperplasia and inflammation occurred in the larynx in all the section levels studied. Multifocal degeneration of the bronchial epithelium was observed in the lungs and squamous metaplasia of the ventral respiratory epithelium and degeneration of Jacobson’s organ were found in section levels I and II in the nose. Also in the trachea, squamous metaplasia was observed both at the tracheal carina and at the ventral epithelium. A NOAEC (no observed adverse effect concentration) for local effects could therefore not be derived, the LOAEC (lowest observed adverse effect concentration) was 3 mg/m^3^. At this concentration, 0.06 ml formaldehyde/m^3^ and no 2-aminoethanol was detected in the vapour phase (BASF SE 2011; Hartwig 2015). Since all animals in the low concentration group displayed effects on the larynx, a benchmark calculation was not possible (Hartwig 2015).

### Genotoxicity

#### In vitro

In two mutagenicity tests in Salmonella typhimurium, *N*,*N′*,*N′′*-tris(β-hydroxyethyl)hexahydro-1,3,5-triazine was not mutagenic; in a third mutagenicity test in Salmonella typhimurium, the substance produced questionably positive results only in the strain TA100 (Hartwig 2015).

*N*,*N′*,*N′′*-Tris(β-hydroxyethyl)hexahydro-1,3,5-triazine did not induce DNA repair synthesis (UDS) in primary rat hepatocytes. The substance yielded negative results in the HPRT assay in V79 cells, but induced an increase in chromosomal aberrations in a concentration-dependent manner in V79 cells without the addition of a metabolic activation system (Hartwig 2015).

#### In vivo

*N*,*N′*,*N′′*-Tris(β-hydroxyethyl)hexahydro-1,3,5-triazine did not induce UDS in the liver of Wistar rats (Hartwig 2015).

In the micronucleus tests with oral, dermal and subcutaneous administration already described in the documentation of 1988, *N*,*N′*,*N′′*-tris(β-hydroxyethyl)hexahydro-1,3,5-triazine did not lead to clastogenic effects (no other details; Henschler 1991). Likewise, the substance did not increase micronuclei in polychromatic erythrocytes of the bone marrow in more recent mouse micronucleus tests with intraperitoneal or oral administration up to toxic doses (Hartwig 2015).

It is also unclear whether, after the administration of formaldehyde, cytogenetic effects can only occur as a result of local exposure or also as a result of systemic availability of formaldehyde (Greim 2002). The negative in vivo test results for *N,N′,N′′*-tris(β-hydroxyethyl)hexahydro-1,3,5-triazine do not, therefore, contradict those for formaldehyde. 2-Aminoethanol, another hydrolysis product, is not genotoxic in vitro and in vivo (Greim 1999).

### Carcinogenicity

There are no long-term studies available for *N*,*N′*,*N′′*-tris(β-hydroxyethyl)hexahydro-1,3,5-triazine. In a subchronic limit test, in which 50 mg of *N*,*N′*,*N′′*-tris(β-hydroxyethyl)hexahydro-1,3,5-triazine per animal was brushed onto the shaved skin of female NMRI mice 3 times per week for 31 weeks, tumour incidences were not increased. Neither did the test substance induce papillomas (Hartwig 2015).

The local carcinogenicity of the hydrolysis product formaldehyde is extensively documented (Greim 2002).

2-Aminoethanol has a tumour-promoting effect on mouse skin, which is presumably due to its irritating effect (Greim 1999). Long-term carcinogenicity studies with 2-aminoethanol are not available.

## Manifesto (MAK value/classification)

The most sensitive end points are the carcinogenic and locally irritating effects of the hydrolysis product formaldehyde.

**Carcinogenicity. **Long-term studies of the carcinogenicity of *N*,*N′*,*N′′*-tris(β-hydroxyethyl)hexahydro-1,3,5-triazine are not available. *N*,*N′*,*N′′*-Tris(β-hydroxyethyl)hexahydro-1,3,5-triazine itself was not found to have genotoxic potential in the available tests except for a questionably positive result in the mutagenicity test in Salmonella typhimurium. However, possible genotoxic effects at the target sites upper respiratory tract and nose (as with formaldehyde) have not been investigated.

The local carcinogenic effects of the hydrolysis product formaldehyde, on the other hand, have been extensively documented (Greim 2002; Hartwig 2014). Formaldehyde is classified in Carcinogen Category 4, as it is carcinogenic in nasal tissues only at concentrations exceeding their detoxification capacity. *N*,*N′*,*N′′*-Tris(β-hydroxyethyl)hexahydro-1,3,5-triazine releases formaldehyde very rapidly (see Section ‟Hydrolysis”). Studies of how much formaldehyde is released in the respiratory tract are not available. The half-life in the nose under physiological conditions (37 °C and a pH of around 7) has not been determined to date. Therefore, the worst case, the sudden complete release of formaldehyde, is assumed for the workplace situation after inhalation and dermal exposure. In addition, the second hydrolysis product, 2-aminoethanol, has a local irritant effect. Due to the local carcinogenicity of formaldehyde, *N*,*N′*,*N′′*-tris(β-hydroxyethyl)hexahydro-1,3,5-triazine could, by analogy, be classified in Carcinogen Category 4. However, as no MAK value can be derived for *N*,*N′*,*N′′*-tris(β-hydroxyethyl)hexahydro-1,3,5-triazine, the substance has been assigned to Carcinogen Category 2 and given the footnote “Prerequisite for Category 4 in principle fulfilled, but insufficient data available for the establishment of a MAK or BAT value”.

**MAK value and peak limitation. **The rate of the hydrolysis of *N*,*N′*,*N′′*-tris(β-hydroxyethyl)hexahydro-1,3,5-triazine is dependent on concentration, pH and temperature, whereby three molecules of formaldehyde and 2-aminoethanol can be formed from each molecule of *N*,*N′*,*N′′*-tris(β-hydroxyethyl)hexahydro-1,3,5-triazine.

An inhalation study with *N*,*N′*,*N′′*-tris(β-hydroxyethyl)hexahydro-1,3,5-triazine in rats is available which reported a stronger local irritant effect (LOAEC 3 mg/m^3^ ≙ 0.33 ml/m^3^) after exposure for 28 days compared with that of formaldehyde and 2-aminoethanol.

For formaldehyde, the NOAEC is 2 ml/m^3^ after subacute inhalation exposure (see documentation “Formaldehyde”; Greim 2002). The NOAEC for 2-aminoethanol is 10 mg/m^3^ (3.95 ml/m^3^) (see documentation “2-Aminoethanol”; Hartwig and MAK Commission 2018) and is thus higher than that of both *N*,*N′*,*N′′*-tris(β-hydroxyethyl)hexahydro-1,3,5-triazine and formaldehyde. The stronger local effect of *N*,*N′*,*N′′*-tris(β-hydroxyethyl)hexahydro-1,3,5-triazine compared with that of formaldehyde and of 2-aminoethanol is probably the result of aerosol impaction. The calculated vapour pressure of *N*,*N′*,*N′′*-tris(β-hydroxyethyl)hexahydro-1,3,5-triazine is 5 × 10^–8^ hPa, which corresponds to a vapour saturation concentration of 0.005 ml/m^3^ (note: the measured vapour pressure of 0–0.006 hPa given in Hartwig (2015) is not valid, as the vapour pressure of the individual components was determined, not that of the triazine). Formaldehyde or 2-aminoethanol itself would theoretically both be present in vapour form at the LOAEC (3 mg/m^3^) of *N*,*N′*,*N′′*-tris(β-hydroxyethyl)hexahydro-1,3,5-triazine. However, since only 0.06 ml formaldehyde/m^3^ and no 2-aminoethanol were detected in the vapour phase at 3 mg triazine/m^3^ (BASF SE 2011), hydrolysis of the triazine to form these components apparently occurs only after reaching the aqueous environment in the respiratory tract. Due to the strong effects of *N*,*N′*,*N′′*-tris(β-hydroxyethyl)hexahydro-1,3,5-triazine at the LOAEC of the 28-day study and the lack of a NOAEC, a MAK value cannot be derived from this study (see Hartwig 2015). Due to the role of aerosol impaction, a MAK value cannot be established in analogy to that for the hydrolysis product formaldehyde, either. Peak limitation is therefore not applicable.

When used in dilute aqueous solutions, complete hydrolysis should be expected and therefore the MAK values for formaldehyde (Greim 2002; Hartwig 2014) and 2-aminoethanol (Greim 1999; Hartwig and MAK Commission 2018) should be observed.

**Absorption through the skin. **There are no experimental data available for the absorption of *N*,*N′*,*N′′*-tris(β-hydroxyethyl)hexahydro-1,3,5-triazine through the skin. For model calculations according to IH SkinPerm v2.04 (Tibaldi et al. 2014) and Fiserova-Bergerova et al. (1990), a substance concentration of 1% in aqueous solution was assumed with a view to minimizing the irritation and sensitization potential of mixtures (see Hartwig 2015; Henschler 1991). For standard conditions (60 minutes exposure duration, 2000 cm^2^ of exposed skin), an absorbed amount of about 9 and 22 μg/kg body weight, respectively, can thus be estimated. These results are far below the NOAEL values for systemic toxicity from an oral 90-day study in rats (64 and 91 mg/kg body weight, respectively), in which decreased body and organ weights were determined as end points (Hartwig 2015). Therefore, *N*,*N′*,*N′′*-tris(β-hydroxyethyl)hexahydro-1,3,5-triazine is not designated with an “H” (for substances which can be absorbed through the skin in toxicologically relevant amounts).

This applies also in view of the possibly rapid systemic release of formaldehyde in the bloodstream: for a person of 70 kg, the maximum estimated total absorbed amount of *N*,*N′*,*N′′*-tris(β-hydroxyethyl)hexahydro-1,3,5-triazine under the above-mentioned conditions is 1.54 mg (22 µg/kg body weight × 70 kg) (0.007 mmol). Assuming rapid complete hydrolysis, this results in the release of 0.021 mmol (0.6 mg) formaldehyde. The physiological level of formaldehyde in human blood is about 2–3 mg/l (Heck et al. 1985) and thus 10–15 mg in 5 l blood, so that the maximum additional contribution of 0.6 mg formaldehyde is within the variation range of physiological exposure.

**Germ cell mutagenicity. ***N*,*N′*,*N′′*-Tris(β-hydroxyethyl)hexahydro-1,3,5-triazine is not mutagenic in vitro in bacteria and mammalian cells. The induction of chromosomal aberrations in vitro has not been confirmed in several micronucleus tests with oral, dermal or intraperitoneal administration. A UDS test in vivo also yielded a negative result. Thus, in studies with *N*,*N′*,*N′′*-tris(β-hydroxyethyl)hexahydro-1,3,5-triazine alone, there was no evidence of genotoxic effects. Studies in germ cells are not available. Assuming that after inhalation all formaldehyde is rapidly released in the upper respiratory tract via hydrolysis, systemic availability of the substance seems unlikely. However, data for this are not available.

Formaldehyde is classified in Category 5 for germ cell mutagens. This means that, if the MAK value of 0.3 ml/m^3^ is observed, inhalation exposure to formaldehyde is expected to make only a very small contribution to the genetic risk for humans (Greim 2002; RAC and SEAC 2020).

Theoretically, by analogy with formaldehyde,* N*,*N′*,*N′′*-tris(β-hydroxyethyl)hexahydro-1,3,5-triazine could be classified in Category 5 for germ cell mutagens. However, a MAK value cannot be established for *N*,*N′*,*N′′*-tris(β-hydroxyethyl)hexahydro-1,3,5-triazine. Since data for the systemic bioavailability of *N*,*N′*,*N′′*-tris(β-hydroxyethyl)hexahydro-1,3,5-triazine and the formaldehyde released by hydrolysis are not available, there is no experimental evidence that the released formaldehyde reaches the germ cells in active form. Therefore, *N*,*N′*,*N′′*-tris(β-hydroxyethyl)hexahydro-1,3,5-triazine has been classified in Category 3 B for germ cell mutagens.
